# Opportunities for Characterizing Geological Flows Using Magnetic Resonance Imaging

**DOI:** 10.1016/j.isci.2020.101534

**Published:** 2020-09-05

**Authors:** Einat Lev, Christopher M. Boyce

**Affiliations:** 1 Lamont-Doherty Earth Observatory, Columbia University, Palisades, NY 10964, USA; 2 Department of Chemical Engineering, Columbia University, New York, NY 10027, USA

**Keywords:** Chemical Engineering, Earth Sciences, Methods in Earth Sciences, Physics, Engineering

## Abstract

Geological flows—from mudslides to volcanic eruptions—are often opaque and consist of multiple interacting phases. Scaled laboratory geological experiments using analog materials have often been limited to optical imaging of flow exteriors or *ex situ* measurements. Geological flows often include internal phase transitions and chemical reactions that are difficult to image externally. Thus, many physical mechanisms underlying geological flows remain unknown, hindering model development. We propose using magnetic resonance imaging (MRI) to enhance geosciences via non-invasive, *in situ* measurements of 3D flows. MRI is currently used to characterize the interior dynamics of multiphase flows, distinguishing between different chemical species as well as gas, liquid, and solid phases, while quantitatively measuring concentration, velocity, and diffusion fields. This perspective describes the potential of MRI techniques to image dynamics within scaled geological flow experiments and the potential of technique development for geological samples to be transferred to other disciplines utilizing MRI.

## Graphical Abstract

**Figure:**
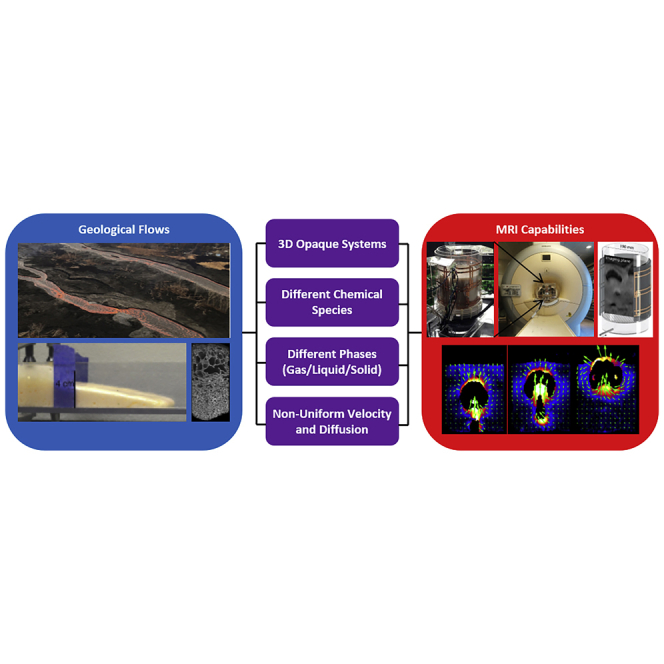

## Introduction

Most people are familiar with the use of magnetic resonance imaging (MRI) to investigate the interior of the human body for medical purposes; however, many scientists are unaware of the use of MRI to study flow and reactions in analog chemical reactors. Physicists, chemists, and engineers currently use MRI to study multiphase flows and chemical reactions. However, many of these MRI experts and users may not be fully aware of the parallel systems found in geological flows, as well as the current open questions and techniques used to study these flows. This perspective seeks to provide insights to geoscientists on the capabilities of MRI to image opaque multiphase flows and reactions in 3D as well as inform MRI specialists on the current gaps in techniques and knowledge in geological flows that can be addressed using MRI. We begin with an overview of current measurement techniques used to study geological flows in the laboratory, highlighting knowledge gaps arising from limitations on penetration depth and contrast. We then provide background on the fundamental science, capabilities, and limitations of MRI, to further familiarize geoscientists with MRI principles and procedures. The third section of the paper focuses on the current uses of MRI to study multiphase flows and reactions, many aspects of which mirror capabilities needed to study geological flows. We conclude with our perspective on how MRI has the potential to greatly enhance the toolkit available for studying geological flows to, in conjunction with modeling and other experimental techniques, generate new knowledge with important applications. Overall, we anticipate that not only will MRI create new insights into geological flows but also the new and unique samples studied by geoscientists will lead to MRI technique development that can be adopted by the medical, physics, chemistry, and engineering communities.

## Current Measurement Techniques Used to Study Geological Flows

Many geological and natural environments involve multiphase flows. Magma, comprising solid particles, gas bubbles, and liquid silicate melt, is a clear example of a three-phase suspension. Gas hydrates and submarine gas seeps are another, as the gas bubbles interact with liquid water and the rock matrix. Two-phase flows are even more ubiquitous, and solid-liquid, solid-gas, and liquid-gas scenarios are all common. Sediments carried by rivers, and water, pollutants, and metallic ore concentrates percolating through a porous rock, are examples of geological solid-liquid interactions. The migration of melt through a partially molten rock matrix to pool into high melt fraction reservoirs and potentially be extracted to the surface is a complex reactive flow process involving chemical reactions and liquid-solid interactions. There are many examples of gas-solid interaction, for instance, clouds of aerosols such as volcanic ash, anthropogenic particulate matter, or dust interaction with the atmosphere. Geological sequestration of CO_2_, a potential tool for reducing anthropogenic impact on climate, involves the reactive flow of gas through rocks and is impacted by both chemical reactions and fluid mechanics. Hydrothermal systems such as geysers and submarine gas chimneys exhibit gas-liquid interaction.

Geoscientists use analytical and numerical models to study the multiphase interactions at the particle or bubble level as well as the system's behavior at large. For example, forecasting volcanic eruptions relies on models that capture the dynamics of magma ascending within a conduit, including its deformation, fragmentation, and bubble coalescence. Other models, used to forecast the impact of volcanic eruptions on their surroundings, rely on understanding of lava dynamics at the microscale of bubbles and particles and their impact on large-scale processes of channel formation, solidification, and inflation. Such models must be tested and constrained by observations. However, the time and length scale of many geological processes, and often their inaccessibility, make direct observation and measurements of these processes impractical or impossible. It is, therefore, common practice in geoscience to use scaled (“analog”) laboratory experiments with materials and geometries analogous to the geological materials and processes (Kavanagh et al., 2018). Sedimentologists often study deposition and erosion processes in a laboratory flume (e.g., Baar et al., 2018; Pohl et al., 2020). Volcanologists use analog experiments to study all the various components of the magmatic system, from the magma reservoir (Galland et al., 2014) and plumbing system (Taisne et al., 2011) to eruptions and their products (Del Bello et al., 2017; Iverson et al., 2010; Lev et al., 2019; Lube et al., 2015).

Geoscientists performing analog experiments pay special attention to how the experiments scale compared with the natural phenomena of interest. This is commonly achieved by considering a set of non-dimensional parameters that express the ratio between the forces and length- and timescales within the analog and natural system. For instance, to study the emplacement of lava flows in the laboratory, scientists use materials with viscosities and thermal properties that yield behavior within the same regime (e.g., turbulent versus laminar, solidification dominated versus advection dominated) as the natural system (e.g., Gregg and Fink, 2000; Kerr et al., 2006). Sugar-based syrups, clay slurries, silicone oil, and polyethylene glycol wax have all been utilized as analogs for lava and magma. To study processes such as tectonic plate deformation or dyke intrusion, where elasticity plays a role, scientists choose materials that have a similar relative importance of elastic and plastic behavior as that of the rocks or tectonic plates in which they are interested. Geologists interested in reactive flows such as magma infiltration through the crust have used analog systems such as salt (NaCl) and water (Kelemen et al., 1995) and discovered the flow focuses into channels. These findings have since been used in models of melt extraction under mid-oceanic ridges (e.g., Spiegelman and Kelemen, 2003) and other reactive flows (e.g., Kang et al., 2010). Sediment transport being studied in laboratory hydrographs uses materials similar to the natural system (water and rock particles) and thus scaling to the natural system comes from selecting the appropriate length- and timescales by adjusting slopes, thickness, and aspect ratio, and peak discharge rates (Yager et al., 2015). Bubble behavior is scaled through the capillary number *Ca*, which expresses the ratio between shear forces and surface tension forces (Manga et al., 1998; Truby et al., 2015), and the aim is to have a similar regime (*Ca* > 1 or *Ca* < 1) in the laboratory as in the natural system being studied. Analog experiments have also helped constrain the importance of parameters such as particle shape and size distribution by mimicking, and also systematically expanding upon, natural systems (e.g., Cimarelli et al., 2011; Gomez, 1994; Moitra and Gonnermann, 2015).

**Figure 1:**
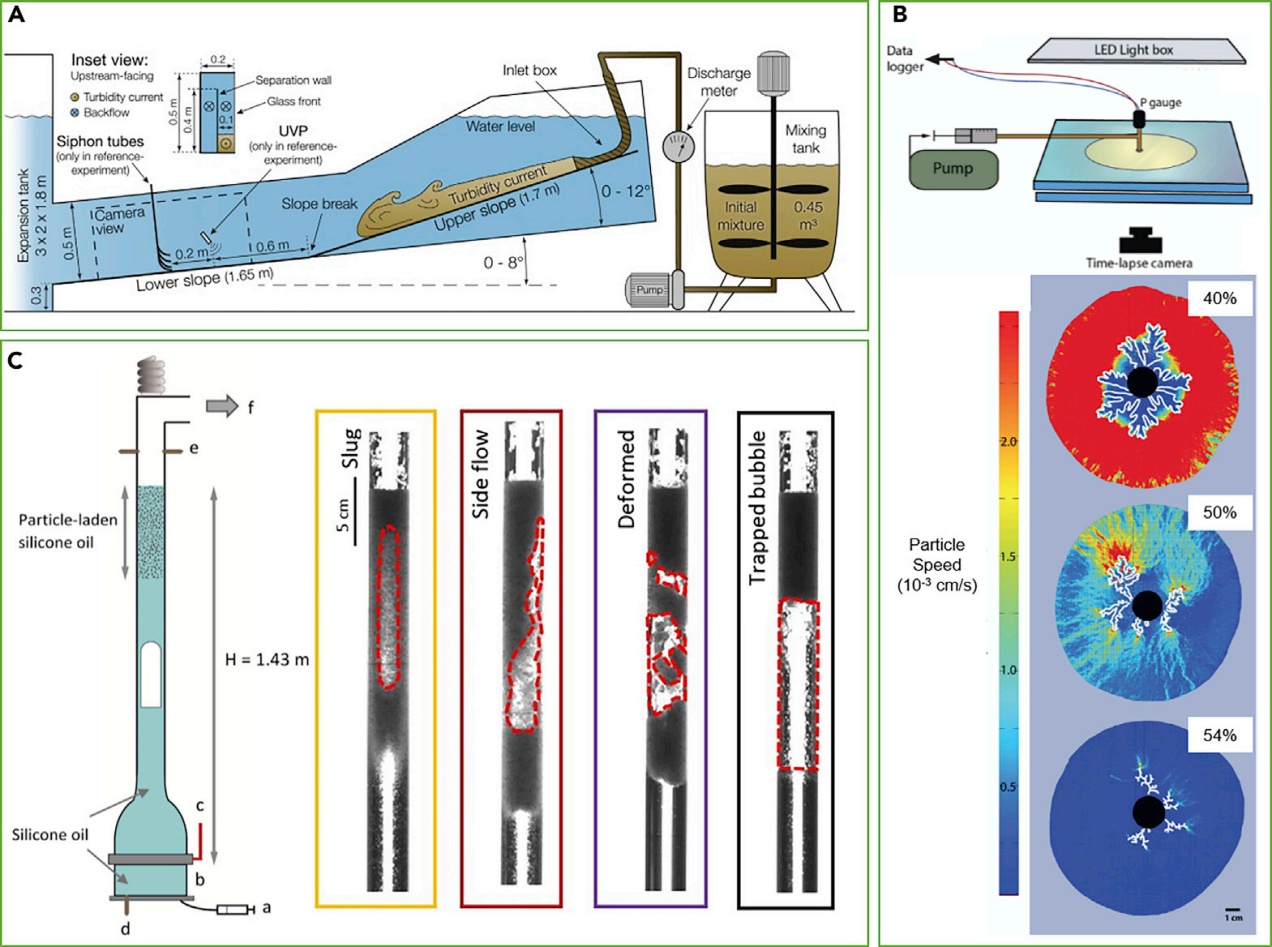
Examples of Analog Experiments Simulating Geological Multiphase Flows, Showing Experimental Setups and Resulting Images and Measurements (A) Experimental setup of a flume experiment (Pohl et al., 2020), where a mixture of sediments and fresh water is released into a water tank. Velocity is measured using an ultrasonic velocity probe (UVP) positioned above the flow. Flume experiments are a common tool in the study of turbidity channels and river dynamics. (B) Experimental setup and particle image velocimetry measurements from bubble injection experiments using a small-gap parallel-plate geometry to study the development of permeable pathways in a particle-rich suspension with varying particle fraction (Oppenheimer et al., 2015). Labels give particle concentration in percentages. (C) Experimental setup and snapshots from conduit flow experiments where a gas slug rises within a vertical tube of silicone oil and interacts with a particle-laden cap to simulate ascent of gas slugs in a magmatic conduit (Oppenheimer et al., 2020).

Imaging is a critical component of analog experiments, as it provides the direct observations of what the system is doing and how it responds to forcing. For some experiments, it is feasible to select analog materials and setups that are sufficiently transparent and allow imaging of the interior. However, for most scenarios this is not the case. Multiphase analogs, similar to the original geological suspensions they simulate, are often opaque, a result of the suspended bubbles and particles. Scientists are thus limited in their ability to directly witness the dynamics of flow. Many experiments, particularly of deformation and reactive flows, are limited to measuring the products only at the end of the experiment (e.g., Kelemen et al., 1995; Liu et al., 2012; Peuble et al., 2015). When opaque experiments are imaged from the outside, viewers are limited to observing only the part of the flow that interacts with the walls, which is not necessarily representative of the flow's interior (see Figures 1A and 1C). Analysis of the velocity of the fluid using observations from the outside is also limited, as only motion in the observed plane can be made, while inward and outward components of the motion must be inferred. In many experiments, viewing of the entire flow volume was achieved by making experimental setups quasi-two dimensional, for instance, Hele-Shaw cells (see Figure 1B) or long and narrow tubes. Again, such experiments are heavily influenced by the interaction of the fluids with the cell's walls, and translation of the results to three-dimensional natural systems may not be straight forward. When dynamics in the interior of the flow are the key observation to be made, as is the case for sedimentological flume experiments, scientists use tools such as acoustic Doppler velocity meters to measure velocity and sediment concentrations. Although useful, such tools provide only a local measurement at a specific point and not a full picture of the entire flow.

A new and promising approach has been imaging multiphase experiments using X-ray micro-computer tomography (CT). This powerful imaging technique relies on density differences between the liquid, gas, and solid in the experiment and can also detect cracks. However, experiments are limited in size to a few centimeters, owing to scanner dimensions, and in the speed of flow they can capture. For instance, Zheng et al. (2019) performed a reactive flow experiment in which water percolated through a porous rock sample (a cylinder 5 mm in height an 2.3 mm in diameter) and the reaction induced fracturing of the sample. By performing the experiment within a powerful synchrotron facility, they achieved high spatial resolution (6.5 μm^3^ voxels) but each volumetric scan took 180 s. Pistone et al. (2015) used a synchrotron X-ray source as well to study gas-driven melt segregation in magmatic mush. They achieved a spatial resolution of under 3 μm/pixel side, but it took 8 s to image the 1 mm^3^ sample. Less powerful X-ray CT scanners have been used to image faults in a deforming plate-boundary analog (Adam et al., 2013), where resolution and speed were not as critical. A notable limitation of X-ray tomography is that it cannot directly measure the velocity of the fluid, and that has to be calculated using post-processing tools such as voxel tracking or image correlation. The promise of tomographic imaging in providing insights into geological flows and the limitations of X-ray measurements create impetus to investigate the potential for MRI to study geological flows.

## Background on MRI

**Figure 2:**
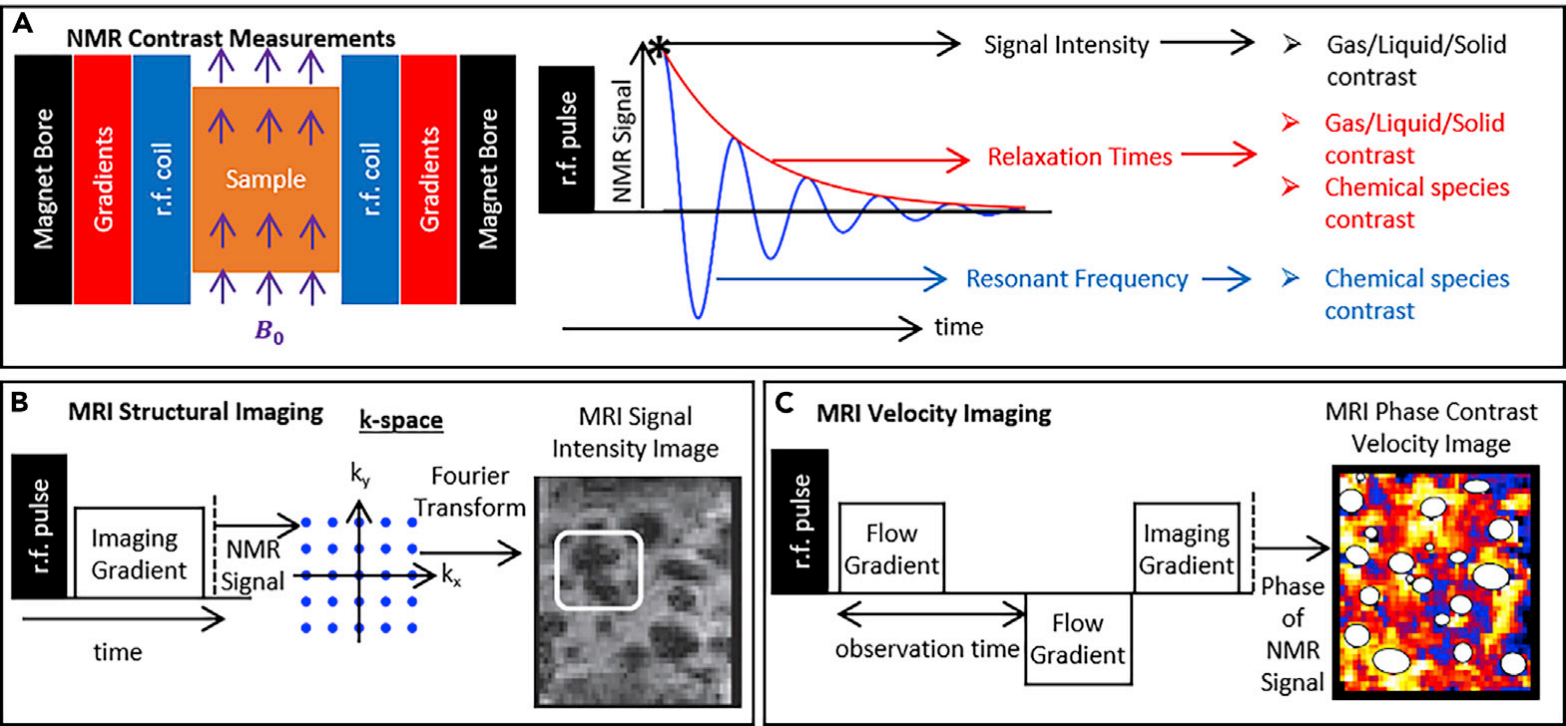
Schematic of Basic MRI Setup, Concepts and Capabilities (A) Schematic of a sample in an NMR system (left) and pulse sequence to receive NMR signal (center) to achieve contrast and images (right) from NMR. Pulse sequences for (B) MRI structural imaging and (C) MRI velocity imaging. Structural and velocity images of gas bubbles rising through liquid are adapted from Tayler et al. (2012a).

MRI (Lauterbur, 1973; Mansfield, 1977) is a non-invasive imaging technique based on nuclear magnetic resonance (NMR) (Bloch, 1946; Purcell, 1946). Broadly speaking, NMR measurements consist of placing a sample in a static magnetic field and using radiofrequency (r.f.) pulses to produce an oscillating r.f. signal from the sample with an amplitude that decays over time (see Figure 2A). The frequency of NMR signal can be used to distinguish between the nuclei generating the signal as well as between chemical species with the same active nuclei. The signal intensity can be used to distinguish between phases (gas, liquid, and solid) as well as quantify concentration. The decay rates in NMR signal amplitude can be related to *relaxation times*, which can distinguish between chemical species and phases. MRI refers to the ability to distinguish between these NMR signals depending on where in 3D space they originate to non-invasively image a 3D sample with the same contrast enabled by NMR. This imaging is enabled by applying spatial magnetic field gradients to the static magnetic field (Figure 2B), enabling NMR signal acquisition in inverse or *k-space*, which can be used to reconstruct an image in real space via a Fourier transform. Pulsing magnetic field gradients in time and separating these pulses by a specified amount of time (Figure 2C) enables measurement of velocity, velocity distribution, and diffusion in the sample (Callaghan, 1991). In such measurements, the phase or intensity of NMR signal relates mathematically to the displacement a nucleus in the sample has undergone in the period between the two pulsed gradients. The principles of flow measurements can be combined with the principles of MRI spatial distinction and NMR contrast to achieve images of flow and concentration that distinguish between phases and chemical species within a single measurement. A variety of books (Callaghan, 1991; Levitt, 2008) and review articles (Britton, 2017; Fukushima, 1999; Gladden and Sederman, 2017, 2013; M. Britton, 2010; V. Koptyug, 2014) provide more information on principles of NMR and MRI, as well as the application of MRI to study chemical reactions and flow.

The temporal resolution of MRI measurements ranges from several milliseconds to several minutes, whereas the spatial resolution ranges from tens of microns to a few millimeters. Sample sizes range from several millimeters to hundreds of millimeters. There are trade-offs between achievable spatial resolution, temporal resolution, contrast, and quantitative accuracy of MRI measurements. Thus, from a resolution standpoint, the capabilities of MRI are modest as compared with optical and X-ray techniques. The advantages of MRI as compared with optical techniques come largely in non-invasive characterization of the interior of opaque 3D systems, whereas the advantages as compared with X-ray techniques lie in contrast between chemical species and the quantification of flow. MRI technology is highly flexible and can be tailored for a specific application. For example, Penn et al. (2017) recently imaged particle velocity and concentration in a gas-solid flow system 190 mm in diameter and 300 mm in height at 55 fps with a spatial resolution of 3 mm.

It is worth noting that NMR and MRI present a number of challenges beyond resolution. While the variety of factors affecting NMR and MRI signal (e.g., relaxation times, motion within the sample, sample heterogeneity) make these techniques highly flexible, these factors also make it such that images from MRI can be difficult to interpret and susceptible to artifacts. These difficulties often make MRI measurements non-quantitative, although carefully designed experiments accounting the various factors impacting NMR signal can enable quantitatively accurate MRI measurements of concentration or flow. Sample size also presents a limitation on MRI measurements, since most superconducting magnet systems used for MRI of multiphase flows have can only accommodate samples up to centimeters in length scale. Ways around this sample size limitation include using medical MRI scanners or lower strength permanent magnets (Eidmann et al., 1996; Perlo et al., 2015); however, these methods often come with significant compromises in spatial resolution and motion resolution. MRI also places constraints on the physical properties of the sample that can be measured. Samples with significant paramagnetic material can create hazards due to magnetic forces; electrically conductive materials, including highly ionic solutions, can lead to artifacts or an inability to measure the interior of the sample. Sufficiently high concentration of non-zero magnetic spin nuclei (e.g., ^1^H, ^13^C, ^19^F, ^23^Na, ^31^P) must be present in the sample in order to generate a reasonable signal-to-noise ratio for MRI. Most liquids contain ^1^H nuclei in high-enough concentrations to produce strong NMR signal, whereas gases usually do not. Although techniques exist for characterizing solids directly (Griffin et al., 2016), these typically have a low signal-to-noise ratio and can require long image acquisition times. Examples of ways of circumventing issues with sample properties include using gases with many NMR-active nuclei per molecule (Prado et al., 1999) or using polarizing gases (Bowers and Weitekamp, 1987; Pavlin et al., 2007) as well as using solid particles filled with liquid (Ehrichs et al., 1995) to characterize granular flows. Conducting measurements at elevated temperature and pressure also presents issues for MRI measurements, since high temperature and pressure pose hazards to potentially damage the MRI hardware (V. Koptyug, 2014). These issues are typically circumvented by using thicker sample containers and active cooling systems (Han et al., 2011; V. Koptyug, 2014), and characterization of a chemical reactor at temperatures up to 300°C and pressures up to 30 bar have been achieved (Gladden et al., 2010).

## Current Uses of MRI to Study Multiphase Flow and Reactions

**Figure 3:**
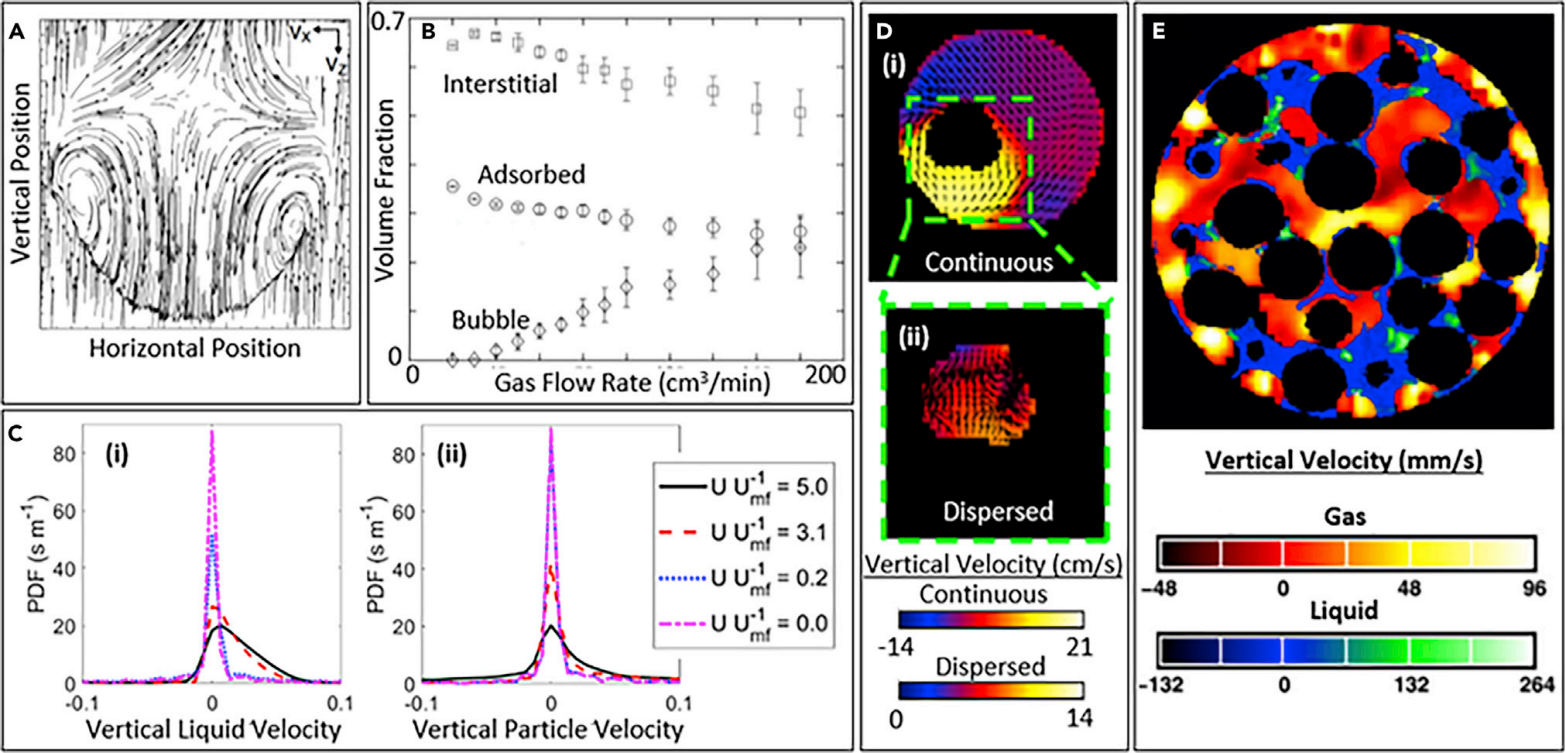
Images Showing Capabilities of MRI to Study Multiphase Flows (A) Arrows showing velocity of liquid in a droplet falling through gas relative to the falling velocity (Han et al., 2001). (B) Quantification of concentration of gas in different phases in a system of granular particles suspended by upward gas flow (Pavlin et al., 2007). (C) Probability density functions (PDFs) of (i) liquid velocity and (ii) particles in a system of granular particles suspended by upward liquid flow at different liquid velocities (U) relative to that needed to suspend the particles (U_mf_) (Boyce et al., 2018). (D) Images of horizontal (arrows) and vertical (colors) velocity of (i) continuous and (ii) dispersed phases in an immiscible liquid-liquid flow with the dispersed phase rising through the continuous phase (Tayler et al., 2014). (E) Image of vertical gas and liquid velocities through a cylinder packed with solid particles (black) (Sankey et al., 2009).

Given the contrast capabilities of NMR, one of the main advantages in using MRI to characterize multiphase flows is the ability to image flow and concentration of multiple phases in the same system. In recent years, MRI has emerged as a powerful tool for characterizing multiphase flow and reactions in the physics, chemistry, and engineering fields. Figure 3 provides examples of ways in which MRI has been used previously to characterize multiphase flows. Most MRI measurements of flow have involved imaging liquid, since liquid has better signal-to-noise ratio than gas and more favorable and tunable relaxation times than solids. Measurements of flow in liquid have included single phase flow in open pipes (Li et al., 1994), the human body (Joseph et al., 2020), and viscometers (Serial et al., 2019); flow through porous media including rock cores (Mitchell et al., 2013) and multiphase flows, such as droplets of water falling through air (Figure 3A) (Amar et al., 2010; Han et al., 2001); flow induced by gaseous bubbles rising through liquid (Tayler et al., 2012b), as well as gas-liquid (Figure 3E) (Sankey et al., 2009) and liquid-liquid (Valiullin and Furó, 2001) flow through porous media. Flows have been measured in laminar (Reci et al., 2018) and turbulent (Kose, 1991; Li et al., 1994) regimes, measuring averaged velocity (Sederman et al., 2004), velocity distribution (Elkins and Alley, 2007), and diffusion tensors (Kärger et al., 1988) as well as diffusion of solutes (Bray et al., 2016). MRI of flow profiles in shearing cells has been used to provide insights on sample rheology; Newtonian fluids as well as shear-thinning (Rofe et al., 1994) and shear-thickening (Fall et al., 2010) suspensions have been studied. Flow velocities measured have ranged from a few μm/s to 10 m/s (V. Koptyug, 2014), measurement of diffusion coefficients greater than 10^−14^ m^2^/s is standard (Gladden and Sederman, 2017) and flow system sizes have ranged from hundreds of micrometers (Zhang and Balcom, 2010) to tens of centimeters (Penn et al., 2017).

Flow through porous media presents difficulties owing to magnetic susceptibility differences between liquid and solids creating unwanted local gradients in the magnetic field (Cotts et al., 1989). However, these issues have been addressed by designing and implementing pulse sequences (Boyce et al., 2016; Cotts et al., 1989) that minimize these effects as well as using additives to match the magnetic susceptibility of liquids and solids (Stoll and Majors, 1982). Acquisition times for many experiments have been in the range of a few to several minutes, preventing temporal resolution of flow fields in unsteady flows. Various strategies have been used to enable effectively instantaneous imaging of unsteady flow profiles. In one study, droplets of liquid were released in a reproducible manner and this release was time-coordinated with MRI measurements to image the flow in a series of identical droplets, ultimately producing an effectively instantaneous image of the flow field in a single droplet (Figure 3A) (Han et al., 2001). In a different study, a rising bubble was suspended in place by downward liquid flow and a rapid imaging pulse sequence was used to generate images of the turbulent flow field at 62 frames per second (Tayler et al., 2012b).

**Figure 4:**
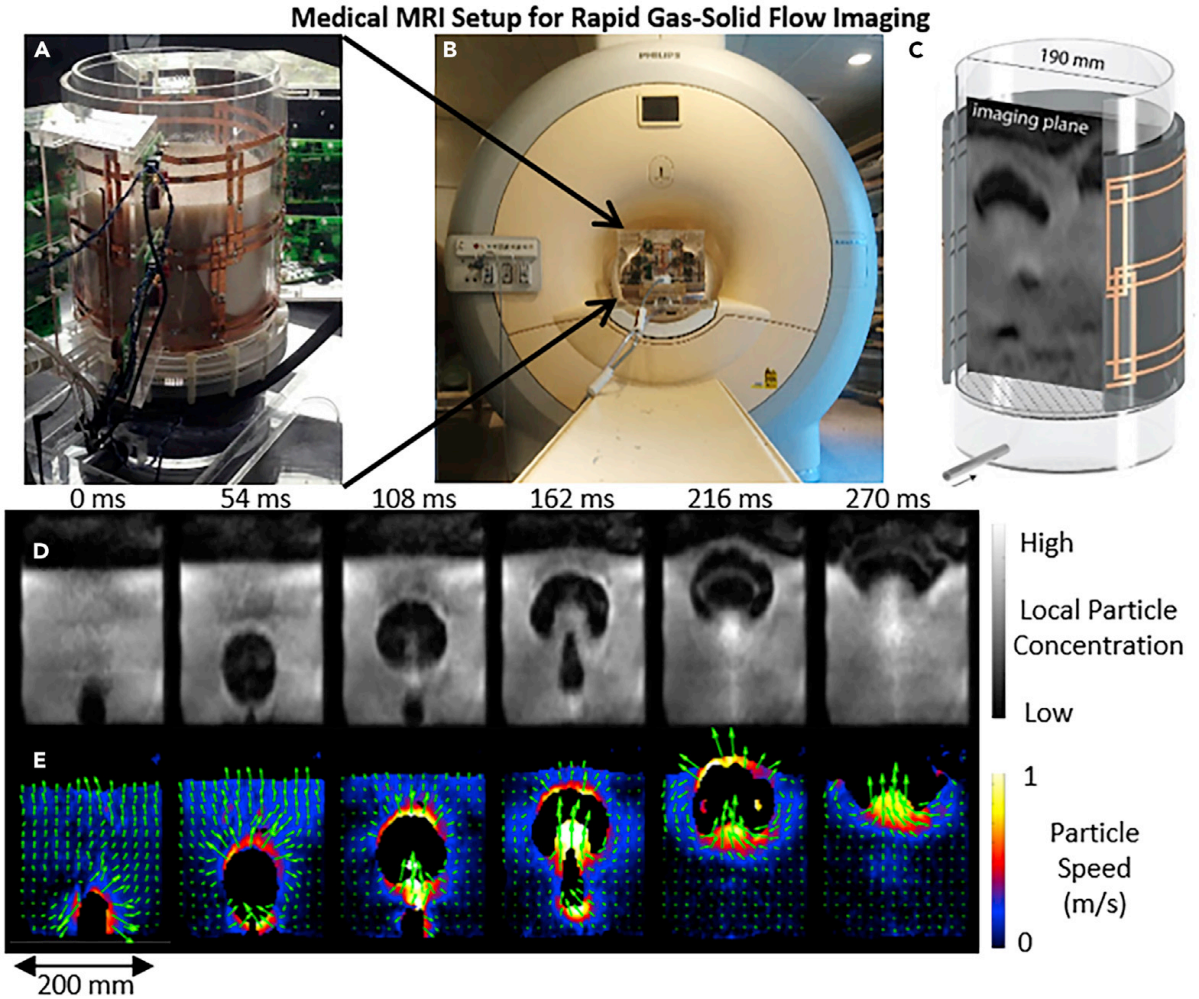
Rapid MRI of Multiphase Flows using a Medical MRI Scanner An experimental setup for studying multiphase flow utilizing (A) a specialized multi-channel r.f. coil placed around cylindrical tank, which were then (B) placed within a medical MRI scanner. (C) A diagram of the r.f. coil and cylinder with a slice showing the imaging plane (Penn et al., 2017). A time series images of the particle concentration (D) and velocity (E) of two bubbles rising through grains and coalescing (Boyce et al., 2019).

Flow of granular particles is also important to a number of physics, chemistry, and engineering problems, and thus techniques have been developed to image granular particles. MRI of granular flows generally involves imaging solid particles containing liquid and receiving NMR signal from the liquid, rather than the solid (Ehrichs et al., 1995). Particles studied include agricultural seeds (Savelsberg et al., 2002), porous particles soaked in liquid (Holland et al., 2010), or specially engineered particles containing liquid (Penn et al., 2017). Experiments have been conducted to image local particle concentration in heterogeneous gas-solid flows (Müller et al., 2006), particle velocity (Holland et al., 2008) and velocity distribution (Savelsberg et al., 2002), local fluctuations in particle velocity (Holland et al., 2008), and diffusion coefficients (Seymour et al., 2000) in fluidized particles. Some studies have also used one type of particles with liquid (“MRI visible”) and another without (“MRI invisible”), so as to characterize mixing between different types of particles under different flow conditions (Hill et al., 1997). Although temporal resolution for these measurements has often been on the order of minutes and excluded insights into instantaneous particle dynamics, a number of solutions have been developed. One study time coordinated the phase of vibration with MRI measurements to characterize flow at a specific point in vibration (Huntley et al., 2007). Other studies have developed and utilized rapid imaging pulse sequences to generate images of heterogeneous particle concentration at acquisition rates as fast as 40 frames per second (Fabich et al., 2016; Müller et al., 2006). A recent study utilized multiple radiofrequency receiver coils, under-sampling of k-space, and rapid imaging pulse sequences to image both particle velocity and concentration at acquisition rates of 55 frames per second (Penn et al., 2017). Figure 4 shows the setup for placing a multi-channel r.f. receiver coil around this gas-solid flow system (Figure 4A), placing this system in a medical MRI scanner (Figure 4B) and imaging particle concentration in a central vertical slice through the cylindrical system (Figure 4C) (Penn et al., 2017). Figure 4D shows images of two gas voids rising through the particles and coalescing over time based on MRI signal intensity imaging, and Figure 4E shows the corresponding particle velocity fields obtained using phase contrast velocimetry (Boyce et al., 2019).

Gas flow has also been studied using MRI. Gases subject to certain types of polarization (e.g., hyperopolarized xenon [Pavlin et al., 2007] and propane subject to parahydrogen induced polarization [Bouchard et al., 2008]) provide enough signal for MRI. Furthermore, gases containing many MRI-active nuclei per molecule (e.g., sulfur hexafluoride) raised to elevated pressure (8 bar) can also provide significant signal (Boyce et al., 2016; Sankey et al., 2009). Experiments have been conducted to extract time-averaged gas velocity and gas concentration in different heterogeneous regions of a gas-solid flow (Figure 3B) (Pavlin et al., 2007). Measurements have provided insights into gas flow through rock cores (Wang et al., 2005), packed particles (Sankey et al., 2009), and fluidized particles (Boyce et al., 2016) as well as turbulent gas flow around obstacles (Gauthier and Newling, 2018; Newling et al., 2004).

Many natural and industrial processes involve multiphase flows coupled with chemical reactions, and the ability of NMR to distinguish between chemical species makes MRI a powerful tool for characterizing such systems. Prior studies have used MRI to image spatial heterogeneities in reaction and changes in reaction extent over time in liquid reactions flowing through porous media based on chemical shift contrast (Gladden et al., 2010). Other studies have investigated propagating chemical species wave fronts in liquid-phase autocatalytic reactions subject to different flow conditions based on changes in relaxation times of different species and pulse sequences designed to distinguish between relaxation times of the different chemical species (Britton, 2006; Koptyug et al., 2003; Serial et al., 2018). Gas-phase reactions catalyzed by solid catalysts have also been imaged utilizing parahydrogen induced polarization, a phenomenon in which hyperpolarized hydrogen reacts with an alkene to produce an alkane, and the alkane product has high MRI signal, yet the reactants have insignificant MRI signal (Bouchard et al., 2008). Furthermore, methods have been developed to use MRI to image local temperature in reacting systems (Zhivonitko et al., 2007).

## Potential for MRI Characterization of Analog Geological Flows

NMR spectroscopy has been utilized extensively in geochemistry and geophysics to detect phase changes and chemical reactions (e.g., Le Losq et al., 2015; Liu et al., 1988; Maekawa and Yokokawa, 1997; Schaller et al., 1992). *In situ* characterization of rock formations and their fluid contents using NMR (“NMR logging”) has been routine for the past few decades in geological exploration of fossil fuels, where it is used to quantify matrix porosity and permeability and to differentiate between types of oil, gas, and water (e.g., Coates et al., 1999). In contrast, MRI, and its spatial imaging power, has seen limited use in geology, with a notable exception of the study of the formation and dissociation of gas hydrates (e.g., Bagherzadeh et al., 2011; Ersland et al., 2010). Aussillous et al. (2006) used MRI's ability to measure simultaneously the concentration, phase, and flow velocity of a sugar-water solution to capture the solidification of a mushy layer cooled from above, an analog for planetary core formation, magma reservoir evolution, and sea ice freezing. Following, Galley et al. (2015) used MRI to capture static images of chimneys and brine inclusions in samples of sea ice. Using a spraying setup within an MRI scanner, Wilbur et al. (2020) imaged the freezing of sea spray, a hazard for off-shore structures and vessels in cold regions. Here, we focus on the potential of flow MRI to study intricate details of laboratory analogs of geological flows, rather than coarser and static measurements in the field.

We postulate that MRI has the potential of enriching and improving our understanding of geological multiphase flows by providing direct observations of the spatial distribution of multiple flow constituents at length scales and timescales that are convenient for scaling between analog experiments and natural flows. For example, experiments examining ascent of a multiphase magma analog in a conduit and performed within an MRI scanner would be able to study a highly vesicular suspension, relevant to many volcanoes, without being limited by its optical opacity. These experiments will be able to approach and investigate the ascent conditions just prior to magma fragmentation and explosion as the MRI will image the fluid speed and concentration at sufficiently fast rates. Conduit shapes in experiments will also not be limited by the need to allow for visual observation from the outside through the conduit wall, and can be made more complex, perhaps more realistic. Such experiments will then be used to test and inform models of volcanic eruptions. Experiments of multiphase gravity currents such as three-phase lava flows, pyroclastic flows, turbidity currents, and mud flows can all benefit from being imaged using MRI. As discussed earlier, natural examples of these flows are difficult to study *in situ* during activity and scaled experiments are often limited to external observations or point measurements internally. MRI will provide unparalleled information on the spatial distribution of phases during the flow at a relevant rate, scanning significant volumes of each flow at rates comparable with flow and circulation speeds. For reactive flows, MRI has the unique power to image the spatial and temporal evolution of the reaction front and the flow velocity at the same time. This can be powerful for analog simulations of magma transport through the crust, and for analog, and even natural, rock-gas compositions, experiments capturing CO_2_ percolating through and reacting with a host rock and enriched liquids migrating through veins and depositing ore metals.

Magma dynamics is a specific application where MRI can provide great insights. The spatial and temporal distribution of bubbles and particles within a magma can change its rheological properties and impact its flow behavior (e.g., Mader et al., 2013; Truby et al., 2015). Yet models of magma ascent in a conduit or of lava flow in a channel usually assume a uniform or a simplified distribution of the three phases through the fluid (e.g., Aravena et al., 2017). These modeled distributions are based on limited experimental data, as described above, or on post-flow observations of the frozen or emptied channels. Models that do not capture magma rheology accurately may miscalculate the eruption volumetric flux, and derived intensity, in response to a given overpressure of the magma chamber, or misestimate the time it will take a lava flow to reach a community downslope. Particularly for lava flows, the interaction of bubbles and solid particles may be different depending on the relative concentrations, and there are likely to be spatial gradients in concentrations as the flow evolves. These interactions, in turn, impact flow evolution, and MRI can provide insight into these processes. Direct measurements of multi-phase spatial distribution through experimental flows analogous to magmatic and other geological flows will thus help improve models and have direct societal impact.

**Figure 5:**
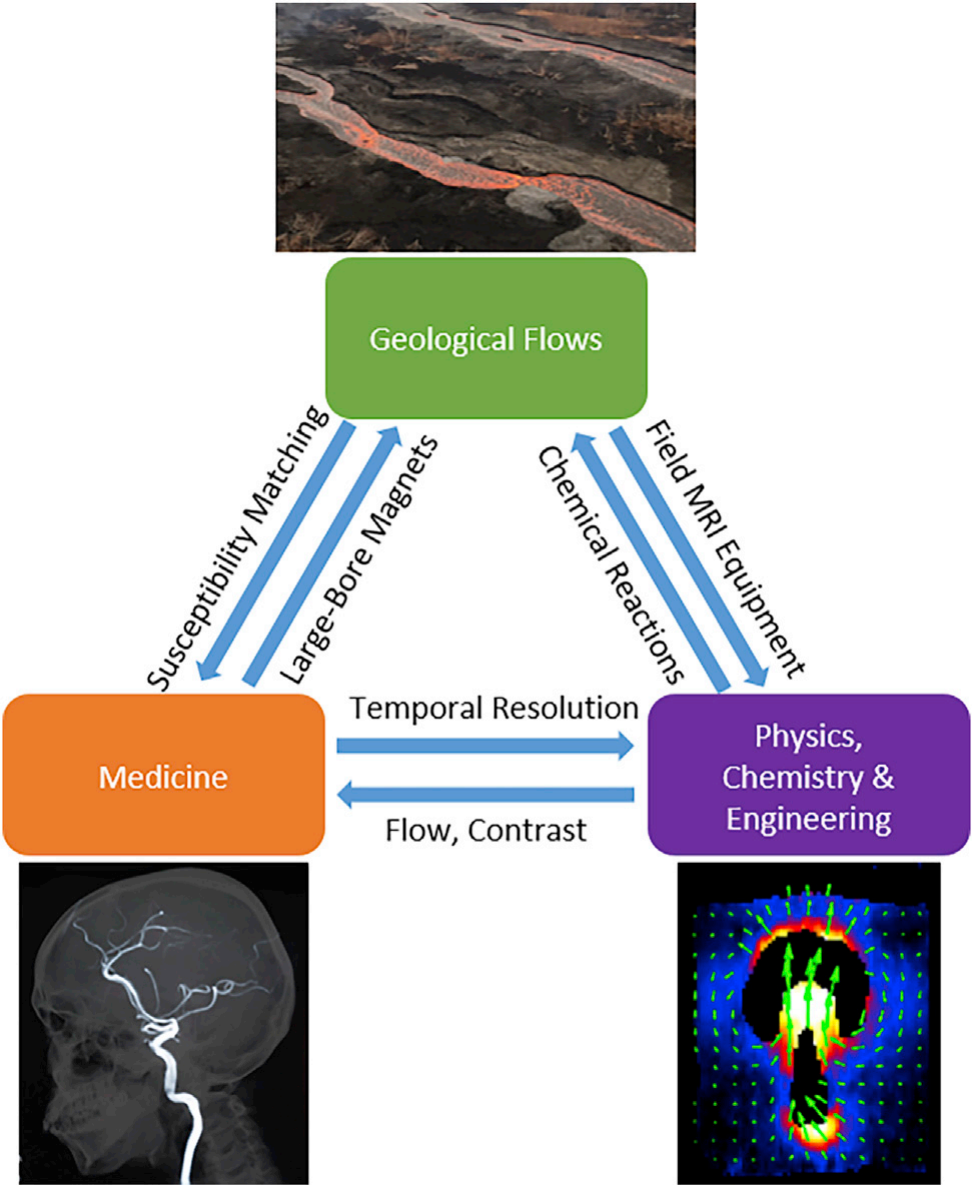
Schematic of Vision for (i) the Medical Community, (ii) the Physics, Chemistry, and Engineering Community, and (iii) the Geosciences Community to Develop Reciprocal Relationships in which MRI Techniques Developed Based on Applications of One Community Are Adopted by Another and Vice Versa Bottom left: Blood flow in the brain imaged by Fathi et al. (2018). Bottom right: Motion and coalescence of bubbles in a granular media imaged by Boyce et al. (2019). Top: Aerial photo of channelized lava flows during the 2018 eruption of Kilauea, Hawai'i, imaged by the USGS. The dish of each channel ranges from 30–100 m, and the lava is approximately 60% vesicular.

Currently, MRI technique developments in the medical community can be applied to the physical sciences community and vice versa, and thus the efforts of both sub-communities strengthen the entire field. The relevance of MRI to solving geological flows could allow for geosciences to become a third sub-community that contributes to overall MRI technique development. For instance, the importance of studying liquid-solid suspensions in geological flows could enable signal enhancement and relaxation time optimization in liquid-solid flows via developing and implementing magnetic susceptibility matching techniques across a range of liquid-solid mixtures. Furthermore, the relevance of flows with multiple types of particles and multiple types of liquids in geosciences could drive the development of rapid, high-fidelity techniques that can distinguish between multiple types of particles and liquids. The reciprocal ways in which different scientific fields with different applications can help each other in MRI technique development are further emphasized in Figure 5. This model has already been seen in the medical and physical sciences communities in which flow and contrast techniques in physical science studies have enabled better measurement of blood flow, whereas temporal resolution development for medical applications have been adopted for faster measurements of gas-solid flows in engineering. We anticipate that this model can be furthered by adding geosciences as a third pillar of applications driving MRI technique development. For example, large-bore magnet development for human anatomy studies in medicine can be used to make better measurements of analog geological flows. Complex, multiphase samples in geological flows will require improvement of magnetic susceptibility matching, which can be used to improve medical studies. Furthermore, development of MRI of chemical reactions in chemistry can be integrated into studies of geological flows. The emphasis of field studies and the wide range of relevant dynamical regimes in geosciences can lead to development of MRI equipment suitable for field studies, which could be applied to MRI studies at engineering plants and a wider range of physics studies.

Overall, we view the capabilities of flow characterization and distinction between phases in 4D of MRI as potentially revolutionary for studying geological flows, and owing to this utility, the geoscience community in turn has an opportunity to push the development of critical new MRI techniques relevant to medicine and physical sciences.

## References

[bib1] Adam J., Klinkmüller M., Schreurs G., Wieneke B. (2013). Quantitative 3D strain analysis in analogue experiments simulating tectonic deformation: integration of X-ray computed tomography and digital volume correlation techniques. J. Struct. Geology.

[bib2] Amar A., Blümich B., Casanova F. (2010). Rapid multiphase flow dynamics mapped by single-shot MRI velocimetry. ChemPhysChem.

[bib3] Aravena Á., de’ Michieli Vitturi M., Cioni R., Neri A. (2017). Stability of volcanic conduits during explosive eruptions. J. Volcanol. Geoth. Res..

[bib4] Aussillous P., Sederman A.J., Gladden L.F., Huppert H.E., Worster M.G. (2006). Magnetic resonance imaging of structure and convection in solidifying mushy layers. J. Fluid Mech..

[bib5] Baar A.W., Smit J.de, Uijttewaal W.S.J., Kleinhans M.G. (2018). Sediment transport of fine sand to fine gravel on transverse bed slopes in rotating annular flume experiments. Water Resour. Res..

[bib6] Bagherzadeh S.A., Moudrakovski I.L., Ripmeester J.A., Englezos P. (2011). Magnetic resonance imaging of gas hydrate formation in a bed of silica sand particles. Energy Fuels.

[bib7] Del Bello E., Taddeucci J., de’ Michieli Vitturi M., Scarlato P., Andronico D., Scollo S., Kueppers U., Ricci T. (2017). Effect of particle volume fraction on the settling velocity of volcanic ash particles: insights from joint experimental and numerical simulations. Sci. Rep..

[bib8] Bloch F. (1946). Nuclear induction. Phys. Rev..

[bib9] Bouchard L.-S., Burt S.R., Anwar M.S., Kovtunov K.V., Koptyug I.V., Pines A. (2008). NMR imaging of catalytic hydrogenation in microreactors with the use of para-hydrogen. Science.

[bib10] Bowers C.R., Weitekamp D.P. (1987). Parahydrogen and synthesis allow dramatically enhanced nuclear alignment. J. Am. Chem. Soc..

[bib11] Boyce C.M., Rice N.P., Ozel A., Davidson J.F., Sederman A.J., Gladden L.F., Sundaresan S., Dennis J.S., Holland D.J. (2016). Magnetic resonance characterization of coupled gas and particle dynamics in a bubbling fluidized bed. Phys. Rev. Fluids.

[bib12] Boyce C.M., Penn A., Pruessmann K.P., Müller C.R. (2018). Magnetic Resonance Characterization of Liquid-Solid Fluidization.

[bib13] Boyce C.M., Penn A., Lehnert M., Pruessmann K.P., Müller C.R. (2019). Magnetic resonance imaging of interaction and coalescence of two bubbles injected consecutively into an incipiently fluidized bed. Chem. Eng. Sci..

[bib14] Bray J.M., Davenport A.J., Ryder K.S., Britton M.M. (2016). Quantitative, in situ visualization of metal-ion dissolution and transport using 1H magnetic resonance imaging. Angew. Chem. Int. Ed..

[bib15] Britton M.M. (2006). Spatial quantification of Mn2+ and Mn3+ concentrations in the Mn-catalyzed 1,4-cyclohexanedione/acid/bromate reaction using magnetic resonance imaging. J. Phys. Chem. A..

[bib16] Britton M. (2010). Magnetic resonance imaging of chemistry. Chem. Soc. Rev..

[bib17] Britton M.M. (2017). MRI of chemical reactions and processes. Prog. Nucl. Magn. Reson. Spectrosc..

[bib18] Callaghan P.T. (1991). Principles of Nuclear Magnetic Resonance Microscopy.

[bib19] Cimarelli C., Costa A., Mueller S., Mader H.M. (2011). Rheology of magmas with bimodal crystal size and shape distributions: insights from analog experiments. Geochem. Geophys. Geosystems.

[bib20] Coates G.R., Xiao L., Prammer M.G. (1999). NMR Logging: Principles and Applications.

[bib21] Cotts R.M., Hoch M.J.R., Sun T., Markert J.T. (1989). Pulsed field gradient stimulated echo methods for improved NMR diffusion measurements in heterogeneous systems. J. Magn. Reson..

[bib22] Ehrichs E.E., Jaeger H.M., Karczmar G.S., Knight J.B., Kuperman V.Y.u., Nagel S.R. (1995). Granular convection observed by magnetic resonance imaging. Science.

[bib23] Eidmann G., Savelsberg R., Blümler P., Blümich B. (1996). The NMR MOUSE, a mobile universal surface explorer. J. Magn. Reson. Ser. A.

[bib24] Elkins C.J., Alley M.T. (2007). Magnetic resonance velocimetry: applications of magnetic resonance imaging in the measurement of fluid motion. Exp. Fluids.

[bib25] Ersland G., Husebø J., Graue A., Baldwin B.A., Howard J., Stevens J. (2010). Measuring gas hydrate formation and exchange with CO2 in Bentheim sandstone using MRI tomography. Chem. Eng. J..

[bib26] Fabich H.T., Sederman A.J., Holland D.J. (2016). Development of ultrafast UTE imaging for granular systems. J. Magn. Reson..

[bib27] Fall A., Lemaître A., Bertrand F., Bonn D., Ovarlez G. (2010). Shear thickening and migration in granular suspensions. Phys. Rev. Lett..

[bib28] Fathi M.F., Bakhshinejad A., Baghaie A., Saloner D., Sacho R.H., Rayz V.L., D’Souza R.M. (2018). Denoising and spatial resolution enhancement of 4D flow MRI using proper orthogonal decomposition and lasso regularization. Comput. Med. Imag. Grap..

[bib29] Fukushima E. (1999). Nuclear magnetic resonance as a tool to study flow. Annu. Rev. Fluid Mech..

[bib30] Galland O., Burchardt S., Hallot E., Mourgues R., Bulois C. (2014). Dynamics of dikes versus cone sheets in volcanic systems. J. Geophys. Res. Solid Earth.

[bib31] Galley R.J., Else B.G.T., Geilfus N.-X., Hare A.A., Isleifson D., Barber D.G., Rysgaard S. (2015). Imaged brine inclusions in young sea ice—shape, distribution and formation timing. Cold Reg. Sci. Technol..

[bib32] Gauthier A.-R.P., Newling B. (2018). Gas flow mapping in a recorder: an application of SPRITE MRI. Appl. Magn. Reson..

[bib33] Gladden L.F., Sederman A.J. (2013). Recent advances in flow MRI. J. Magn. Reson..

[bib34] Gladden L.F., Sederman A.J. (2017). Magnetic resonance imaging and velocity mapping in chemical engineering applications. Annu. Rev. Chem. Biomol. Eng..

[bib35] Gladden L.F., Abegão F.J.R., Dunckley C.P., Holland D.J., Sankey M.H., Sederman A.J. (2010). MRI: operando measurements of temperature, hydrodynamics and local reaction rate in a heterogeneous catalytic reactor. Catal. Today.

[bib36] Gomez B. (1994). Effects of particle shape and mobility on stable armor development. Water Resour. Res..

[bib37] Gregg T.K.P., Fink J.H. (2000). A laboratory investigation into the effects of slope on lava flow morphology. J. Volcanol. Geoth. Res..

[bib38] Griffin J.M., Forse A.C., Grey C.P. (2016). Solid-state NMR studies of supercapacitors. Solid State Nucl. Magn. Reson..

[bib39] Han S.-I., Stapf S., Blümich B. (2001). NMR imaging of falling water drops. Phys. Rev. Lett..

[bib40] Han H., Ouellette M., MacMillan B., Goora F., MacGregor R., Green D., Balcom B.J. (2011). High pressure magnetic resonance imaging with metallic vessels. J. Magn. Reson..

[bib41] Hill K.M., Caprihan A., Kakalios J. (1997). Bulk segregation in rotated granular material measured by magnetic resonance imaging. Phys. Rev. Lett..

[bib42] Holland D.J., Müller C.R., Dennis J.S., Gladden L.F., Sederman A.J. (2008). Spatially resolved measurement of anisotropic granular temperature in gas-fluidized beds. Powder Technol..

[bib43] Holland D.J., Muller C.R., Dennis J.S., Gladden L.F., Davidson J.F. (2010). Magnetic resonance studies of fluidization regimes. Ind. Eng. Chem. Res..

[bib44] Huntley J.M., Martin T.W., Mantle M.D., Shattuck M.D., Sederman A.J., Wildman R.D., Gladden L.F., Halliwell N.A. (2007). NMR measurements and hydrodynamic simulations of phase-resolved velocity distributions within a three-dimensional vibrofluidized granular bed. Proc. Math. Phys. Eng. Sci..

[bib45] Iverson R.M., Logan M., LaHusen R.G., Berti M. (2010). The perfect debris flow? Aggregated results from 28 large-scale experiments. J. Geophys. Res. Earth Surf..

[bib46] Joseph A.A., Voit D., Frahm J. (2020). Inferior vena cava revisited – real-time flow MRI of respiratory maneuvers. NMR Biomed..

[bib47] Kang Q., Lichtner P.C., Viswanathan H.S., Abdel-Fattah A.I. (2010). Pore scale modeling of reactive transport involved in geologic CO2 sequestration. Transp Porous Med..

[bib48] Kärger J., Pfeifer H., Heink W., Waugh J.S. (1988). Principles and application of self-diffusion measurements by nuclear magnetic resonance. Advances in Magnetic and Optical Resonance.

[bib49] Kavanagh Janine L., Engwell Samantha L., Martinl Simon A. (2018). A review of laboratory and numerical modelling in volcanology. Solid Earth.

[bib50] Kelemen P.B., Whitehead J.A., Aharonov E., Jordahl K.A. (1995). Experiments on flow focusing in soluble porous media, with applications to melt extraction from the mantle. J. Geophys. Res. Solid Earth.

[bib51] Kerr R.C., Griffiths R.W., Cashman K.V. (2006). Formation of channelized lava flows on an unconfined slope. J. Geophys. Res. Solid Earth.

[bib52] Koptyug I.V. (2014).

[bib53] Koptyug I.V., Lysova A.A., Parmon V.N., Sagdeev R.Z. (2003). In situ1H NMR imaging study of propagation of concentration waves in an autocatalytic reaction in a fixed granular bed. Kinetics Catal..

[bib54] Kose K. (1991). One-shot velocity mapping using multiple spin-echo EPI and its application to turbulent flow. J. Magn. Reson..

[bib55] Lauterbur P.C. (1973). Image formation by induced local interactions: examples employing nuclear magnetic resonance. Nature.

[bib56] Lev E., Rumpf E., Dietterich H. (2019). Analog experiments of lava flow emplacement. Ann. Geophys..

[bib57] Levitt M.H. (2008). Spin Dynamics: Basics of Nuclear Magnetic Resonance.

[bib58] Li T.-Q., Seymour J.D., Powell R.L., McCarthy K.L., Ödberg L., McCarthy M.J. (1994). Turbulent pipe flow studied by time-averaged NMR imaging: measurements of velocity profile and turbulent intensity. Magn. Reson. Imaging.

[bib59] Liu S.-B., Stebbins J.F., Schneider E., Pines A. (1988). Diffusive motion in alkali silicate melts: an NMR study at high temperature. Geochim. Cosmochim. Acta.

[bib60] Liu F., Lu P., Griffith C., Hedges S.W., Soong Y., Hellevang H., Zhu C. (2012). CO2–brine–caprock interaction: reactivity experiments on Eau Claire shale and a review of relevant literature. Int. J. Greenh. Gas Con..

[bib61] Le Losq C., Mysen B.O., Cody G.D. (2015). Water and magmas: insights about the water solution mechanisms in alkali silicate melts from infrared, Raman, and 29Si solid-state NMR spectroscopies. Prog. Earth Planet. Sci..

[bib62] Lube G., Breard E.C.P., Cronin S.J., Jones J. (2015). Synthesizing large-scale pyroclastic flows: experimental design, scaling, and first results from PELE. J. Geophys. Res. Solid Earth.

[bib63] Mader H.M., Llewellin E.W., Mueller S.P. (2013). The rheology of two-phase magmas: a review and analysis. J. Volcanol. Geoth. Res..

[bib64] Maekawa H., Yokokawa T. (1997). Effects of temperature on silicate melt sructure: a high temperature 29Si NMR study of Na2Si2O5. Geochim. Cosmochim. Acta.

[bib65] Manga M., Castro J., Cashman K.V., Loewenberg M. (1998). Rheology of bubble-bearing magmas. J. Volcanol. Geoth. Res..

[bib66] Mansfield P. (1977). Multi-planar image formation using NMR spin echoes. J. Phys. C Solid State Phys..

[bib67] Mitchell J., Chandrasekera T.C., Holland D.J., Gladden L.F., Fordham E.J. (2013). Magnetic resonance imaging in laboratory petrophysical core analysis. Phys. Rep..

[bib68] Moitra P., Gonnermann H.M. (2015). Effects of crystal shape- and size-modality on magma rheology. Geochem. Geophys. Geosyst..

[bib69] Müller C.R., Davidson J., Dennis J., Fennell P., Gladden L., Hayhurst A., Mantle M., Rees A., Sederman A. (2006). Real-time measurement of bubbling phenomena in a three-dimensional gas-fluidized bed using ultrafast magnetic resonance imaging. Phys. Rev. Lett..

[bib70] Newling B., Poirier C.C., Zhi Y., Rioux J.A., Coristine A.J., Roach D., Balcom B.J. (2004). Velocity imaging of highly turbulent gas flow. Phys. Rev. Lett..

[bib71] Oppenheimer J., Rust A.C., Cashman K.V., Sandnes B. (2015). Gas migration regimes and outgassing in particle-rich suspensions. Front. Phys..

[bib72] Oppenheimer J., Capponi A., Cashman K.V., Lane S.J., Rust A.C., James M.R. (2020). Analogue experiments on the rise of large bubbles through a solids-rich suspension: a “weak plug” model for Strombolian eruptions. Earth Planet. Sci. Lett..

[bib73] Pavlin T., Wang R., McGorty R., Rosen M.S., Cory D.G., Candela D., Mair R.W., Walsworth R.L. (2007). Noninvasive measurements of gas exchange in a three-dimensional fluidized bed by hyperpolarized 129Xe NMR. Appl. Magn. Reson..

[bib74] Penn A., Tsuji T., Brunner D.O., Boyce C.M., Pruessmann K.P., Müller C.R. (2017). Real-time probing of granular dynamics with magnetic resonance. Sci. Adv..

[bib75] Perlo J., Silletta E.V., Danieli E., Cattaneo G., Acosta R.H., Blümich B., Casanova F. (2015). Desktop MRI as a promising tool for mapping intra-aneurismal flow. Magn. Reson. Imaging.

[bib76] Peuble S., Godard M., Luquot L., Andreani M., Martinez I., Gouze P. (2015). CO2 geological storage in olivine rich basaltic aquifers: new insights from reactive-percolation experiments. Appl. Geochem..

[bib77] Pistone M., Arzilli F., Dobson K.J., Cordonnier B., Reusser E., Ulmer P., Marone F., Whittington A.G., Mancini L., Fife J.L., Blundy J.D. (2015). Gas-driven filter pressing in magmas: insights into in-situ melt segregation from crystal mushes. Geology.

[bib78] Pohl F., Eggenhuisen J.T., Cartigny M.J.B., Tilston M.C., de Leeuw J., Hermidas N. (2020). The influence of a slope break on turbidite deposits: an experimental investigation. Mar. Geol..

[bib79] Prado P.J., Balcom B.J., Mastikhin I.V., Cross A.R., Armstrong R.L., Logan A. (1999). Magnetic resonance imaging of gases: a single-point ramped imaging withT1Enhancement (sprite) study. J. Magn. Reson..

[bib80] Purcell E. (1946). Spontaneous emission probabilities at radio frequencies. Physical Review.

[bib81] Reci A., Sederman A.J., Gladden L.F. (2018). Experimental evidence of velocity profile inversion in developing laminar flow using magnetic resonance velocimetry. J. Fluid Mech..

[bib82] Rofe C.J., Lambert R.K., Callaghan P.T. (1994). Nuclear magnetic resonance imaging of flow for a shear-thinning polymer in cylindrical Couette geometry. J. Rheology.

[bib83] Sankey M.H., Holland D.J., Sederman A.J., Gladden L.F. (2009). Magnetic resonance velocity imaging of liquid and gas two-phase flow in packed beds. J. Magn. Reson..

[bib84] Savelsberg R., Demco D.E., Blümich B., Stapf S. (2002). Particle motion in gas-fluidized granular systems by pulsed-field gradient nuclear magnetic resonance. Phys. Rev. E.

[bib85] Schaller T., Dingwell D.B., Keppler H., Knöller W., Merwin L., Sebald A. (1992). Fluorine in silicate glasses: a multinuclear nuclear magnetic resonance study. Geochim. Cosmochim. Acta.

[bib86] Sederman A.J., Mantle M.D., Buckley C., Gladden L.F. (2004). MRI technique for measurement of velocity vectors, acceleration, and autocorrelation functions in turbulent flow. J. Magn. Reson..

[bib87] Serial M.R., Velasco M.I., Maldonado Ochoa S.A., Zanotto F.M., Dassie S.A., Acosta R.H. (2018). Magnetic resonance imaging in situ visualization of an electrochemical reaction under forced hydrodynamic conditions. ACS Omega.

[bib88] Serial M.R., Silletta E.V., Perlo J., Giovacchini J.P., Velasco M.I., Blümich B., Danieli E.D., Casanova F., Acosta R.H. (2019). Single-shot velocity mapping by rewinding of velocity encoding with Echo-Planar Imaging. J. Magn. Reson..

[bib89] Seymour J.D., Caprihan A., Altobelli S.A., Fukushima E. (2000). Pulsed gradient spin echo nuclear magnetic resonance imaging of diffusion in granular flow. Phys. Rev. Lett..

[bib90] Spiegelman M., Kelemen P.B. (2003). Extreme chemical variability as a consequence of channelized melt transport. Geochem. Geophys. Geosystems.

[bib91] Stoll M.E., Majors T.J. (1982). Reduction of magnetic susceptibility broadening in NMR by susceptibility matching. J. Magn. Reson..

[bib92] Taisne B., Tait S., Jaupart C. (2011). Conditions for the arrest of a vertical propagating dyke. Bull. Volcanol..

[bib93] Tayler A.B., Holland D.J., Sederman A.J., Gladden L.F. (2012). Applications of ultra-fast MRI to high voidage bubbly flow: measurement of bubble size distributions, interfacial area and hydrodynamics. Chem. Eng. Sci..

[bib94] Tayler A.B., Holland D.J., Sederman A.J., Gladden L.F. (2012). Exploring the origins of turbulence in multiphase flow using compressed sensing MRI. Phys. Rev. Lett..

[bib95] Tayler A.B., Benning M., Sederman A.J., Holland D.J., Gladden L.F. (2014). Ultrafast magnetic-resonance-imaging velocimetry of liquid-liquid systems: overcoming chemical-shift artifacts using compressed sensing. Phys. Rev. E.

[bib96] Truby J.M., Mueller S.P., Llewellin E.W., Mader H.M. (2015). The rheology of three-phase suspensions at low bubble capillary number. Proc. Math. Phys. Eng. Sci..

[bib97] Valiullin R., Furó I. (2001). Phase separation of a binary liquid mixture in porous media studied by nuclear magnetic resonance cryoporometry. J. Chem. Phys..

[bib98] Wang R., Pavlin T., Rosen M.S., Mair R.W., Cory D.G., Walsworth R.L. (2005). Xenon NMR measurements of permeability and tortuosity in reservoir rocks. Magnetic Resonance Imaging. Magn. Reson. Imaging.

[bib99] Wilbur G., MacMillan B., Bade K.M., Mastikhin I. (2020). MRI monitoring of sea spray freezing. J. Magn. Reson..

[bib100] Yager E.M., Kenworthy M., Monsalve A. (2015). Taking the river inside: fundamental advances from laboratory experiments in measuring and understanding bedload transport processes. Geomorphology.

[bib101] Zhang J., Balcom B.J. (2010). Magnetic resonance imaging of two-component liquid-liquid flow in a circular capillary tube. Phys. Rev. E.

[bib102] Zheng X., Cordonnier B., McBeck J., Boller E., Jamtveit B., Zhu W., Renard F. (2019). Mixed-mode strain Localization generated by hydration reaction at crustal conditions. J. Geophys. Res. Solid Earth.

[bib103] Zhivonitko V.V., Koptyug I.V., Sagdeev R.Z. (2007). Temperature changes visualization during chemical wave propagation. J. Phys. Chem. A..

